# Interactive Effects of Ocean Acidification and Nitrogen-Limitation on the Diatom *Phaeodactylum tricornutum*

**DOI:** 10.1371/journal.pone.0051590

**Published:** 2012-12-07

**Authors:** Wei Li, Kunshan Gao, John Beardall

**Affiliations:** 1 State Key Laboratory of Marine Environmental Science, Xiamen University, Xiamen, China; 2 School of Biological Sciences, Monash University, Clayton, Victoria, Australia; Royal Netherlands Institute of Sea Research (NIOZ), The Netherlands

## Abstract

Climate change is expected to bring about alterations in the marine physical and chemical environment that will induce changes in the concentration of dissolved CO_2_ and in nutrient availability. These in turn are expected to affect the physiological performance of phytoplankton. In order to learn how phytoplankton respond to the predicted scenario of increased CO_2_ and decreased nitrogen in the surface mixed layer, we investigated the diatom *Phaeodactylum tricornutum* as a model organism. The cells were cultured in both low CO_2_ (390 μatm) and high CO_2_ (1000 μatm) conditions at limiting (10 μmol L^−1^) or enriched (110 μmol L^−1^) nitrate concentrations. Our study shows that nitrogen limitation resulted in significant decreases in cell size, pigmentation, growth rate and effective quantum yield of *Phaeodactylum tricornutum*, but these parameters were not affected by enhanced dissolved CO_2_ and lowered pH. However, increased CO_2_ concentration induced higher rETR_max_ and higher dark respiration rates and decreased the CO_2_ or dissolved inorganic carbon (DIC) affinity for electron transfer (shown by higher values for K_1/2 DIC_ or K_1/2 CO2_). Furthermore, the elemental stoichiometry (carbon to nitrogen ratio) was raised under high CO_2_ conditions in both nitrogen limited and nitrogen replete conditions, with the ratio in the high CO_2_ and low nitrate grown cells being higher by 45% compared to that in the low CO_2_ and nitrate replete grown ones. Our results suggest that while nitrogen limitation had a greater effect than ocean acidification, the combined effects of both factors could act synergistically to affect marine diatoms and related biogeochemical cycles in future oceans.

## Introduction

Rising atmospheric CO_2_ concentrations enhance its absorption into the world's oceans, which currently accounts for removal of nearly one third of anthropogenic CO_2_ emissions from the atmosphere [1]. Atmospheric CO_2_ concentrations are expected to reach 800–1000 ppmv by the end of this century according to the “business as usual” CO_2_ emission scenario [2]. Dissolution of CO_2_ into seawater has already induced a global drop in pH of 0.1 units since the end of the Industrial Revolution, and values are expected to drop another 0.3–0.4 units by the end of this century. This decline in pH driven by increased CO_2_ is termed ocean acidification (OA) [3]. The decrease in seawater pH is a consequence of changes in marine chemistry, where increased dissolved CO_2_ leads to increases in H_2_CO_3_ and hence to increases in H^+^ and HCO_3_^−^ concentrations and decreased CO_3_^2−^ concentration and CaCO_3_ saturation state. Changes in pH also affect biogeochemical processes such as alterations to trace metal speciation, which can have significant biological effects [4], [5].

If the photosynthesis of marine eukaryotic phytoplankton were supported solely by the diffusional supply of CO_2_ to the active site of the CO_2_ fixing enzyme Rubisco, then this process would be severely limited at the concentrations of CO_2_ currently found in seawater (<10–30 μmol) [6]. However, most algae have been shown to be able to make extremely efficient use of low levels of dissolved inorganic carbon (DIC) by virtue of inducible carbon concentrating mechanisms (CCMs) [7]. The CCMs act to maintain internal CO_2_ concentrations higher than can be accounted for by diffusion-mediated entry of inorganic carbon.

CCM activity is down-regulated under high CO_2_; enhanced CO_2_ availability could thus reduce the energy cost for CO_2_ transport [8], and the re-allocation of energy may play a critical role in modulating primary production as well as elemental stoichiometry and species composition [6]. This, however, also may depend on other environmental factors. The effects of ocean acidification can have positive, neutral or negative aspects depending on the physiological processes involved, and may be species-specific [9]. While increased primary production under high CO_2_ has been found in many studies [10], energy loss due to enhanced respiration has also been reported under high CO_2_/low pH conditions [11], possibly due to enhanced energy demand associated with the need to maintain intracellular acid-base stability [12]. The effects of ocean acidification are controversial with contradictory trends reported in the literature. While some of this could be due to species-specific responses it could also result from interactive effects with other environmental factors [13], [14].

Nutrient availability is well known to affect algal growth and production. This is especially so for nitrogen availability, which is seen in many cases as a major limiting factor for algal growth in the oceans [15]. Marine phytoplankton may experience increased nutrient limitation in the euphotic layer in the future due to intensified stratification in a warming ocean [16], [17]. Ocean acidification, at the same time, may affect ion and nutrient assimilation of algae either directly by altering proton or ion channels or indirectly by changes in chemical speciation and nutrient availability [18], [19]. Thus, ongoing ocean acidification together with intensified stratification could further decrease marine nutrient availability and uptake rates. Decreased nitrogen availability is expected to lead to decreased synthesis of chlorophyll and proteins in algae, which would have a strong influence on photosynthesis and physiological performance. Nitrogen limitation is known to affect carbon fixation because of potential impacts on levels of Rubisco and other proteins and also because nitrate assimilation is energy dependent and will compete with carbon fixation for ATP and reductant [20]. Thus changes in C and N acquisition may be reflected in altered cell carbon and nitrogen contents [21].

While the impacts of nutrient limitation under present day CO_2_ are well understood and there is an increasing literature on the effects of elevated CO_2_ on phytoplankton physiology and ecology (see reviews by Beardall et al., Riebesell and Tortell and references therein) [10], [22] and elemental ratios [23], most studies on the effects of ocean acidification have been carried out under nutrient replete conditions [11], [24] and there is very little information on interactive effects between nutrient limitation and elevated CO_2_ [10]. Since elevated CO_2_ and ocean acidification in a future world is likely to go hand-in-hand with a more restricted nutrient supply in the low- to mid-latitude open ocean, this is an important issue that needs addressing.

Thus, this paper considers how phytoplankton responses to ocean acidification may be affected by nitrogen limitation. Specifically, we have measured the cell size, growth, pigmentation, quantum yield, respiration, and CCM activity as well as cell carbon and nitrogen contents of *Phaeodactylum tricornutum* grown under high CO_2_ and low nitrogen conditions, to determine the possible interactive effects of ocean acidification and nitrogen limitation on this model diatom species.

## Materials and Methods

### Statement of ethics

The strain of the diatom *Phaeodactylum tricornutum* Bohlin (strain CCMA 106), originally isolated from the oligotrophic waters of the South China Sea in 2004, was obtained from the Center for Collections of Marine Bacteria and Phytoplankton (CCMBP) of the State Key Laboratory of Marine Environmental Sciences (Xiamen University). No specific permits were required for using this species.

### Algal culture conditions

The diatom *Phaeodactylum tricornutum* Bohlin (strain CCMA 106) were grown in artificial seawater with Aquil medium enrichment [25] except that the NO_3_^−^ concentration was adjusted to 110 µmol L^−1^ NO_3_^−^ (HN) or 10 µmol L^−1^ NO_3_^−^ (LN). The nitrogen-limiting level of 10 µM was based on the surface inorganic nitrogen concentrations (unpublished) obtained from the oligotrophic South China Sea, ranging from 0 (undetectable) to 20 µM. Cultures were continuously aerated with ambient air of 390 μatm of CO_2_ (LC) or with high CO_2_ of 1000 μatm (HC) within plant CO_2_ chambers (HP1000G-D, Ruihua Instrument & Equipment Co. Ltd, China) and bubbled at a constant flow rate of 300 ml min^−1^. This allowed the following treatments, combining different N and CO_2_ levels, to be performed: LC-HN, LC-LN, HC-HN, HC-LN. The cells were grown semi-continuously at 20^°^C under 70 μmol photons m^−2^ s^−1^ illumination with a 12L: 12D photoperiod. Dilutions were carried out every 24 h to ensure cell concentrations did not exceed 3×10^5^ cells ml^−1^ at their exponential growth phase so that pH change during growth at each CO_2_ level was less than 0.02 (Table 1). Cells were acclimated to each NO_3_^−^ and CO_2_ combination for more than 10 generations before being used in the experiments described below.

**Table 1:** Chemical parameters of seawater carbonate system.

	pCO_2_	pH_NBS_	DIC	HCO_3_^−^	CO_3_^2−^	CO_2_	TA
	(μatm)		(μmol Kg^−1^)	(μmol Kg^−1^)	(μmol Kg^−1^)^−^	(μmol Kg^−1^)	(μmol Kg^−1^)
LC-HN	416.6±15.4^a^	8.17±0.02^a^	2063.6±13.4^a^	1851.1±12.0^a^	199.1±6.7^a^	13.1±0.1^a^	2349.3±19.1^a^
LC-LN	404.0±11.9^a^	8.18±0.01^a^	2052.4±8.3^a^	1837.2±5.8^a^	202.2±5.8^a^	13.1±0.4^a^	2343.4±15.2^a^
HC-HN	980.4±52.6^b^	7.85±0.02^b^	2209.7±19.3^b^	2072.0±20.8^b^	106.1±3.7^b^	30.9±1.0^b^	2341.4±14.1^a^
HC-LN	952.8±27.5^b^	7.86±0.01^b^	2200.8±11.5^b^	2062.1±9.9^b^	108.0±3.4^b^	30.8±0.9^b^	2336.2±15.1^a^

Parameters of the seawater carbonate system under the ambient (390 μatm, LC) and elevated (1000 μatm, HC) CO_2_ concentrations as well as nitrogen-replete (HN) and limited (LN) conditions before the partial renewal of the medium for the semi-continuous cultures. Total inorganic carbon (DIC), pH, salinity, nutrient concentration and temperature were used to derive all other parameters using the CO_2_ system analyzing software CO2SYS [36]. Data are the means ± SD of 4 measurements. Different letters indicate significant differences among the treatments at the P<0.05 level.

The pH of cultures was measured daily, prior to dilution, with a pH probe (Mettler Toledo DL15 Titrator, Sweden), which was calibrated with standard NBS (National Bureau of Standards) buffer solutions (Hanna) at three pH points (pH 10.01, pH 7.01 and pH 4.01). Measurement of dissolved inorganic carbon (DIC) was carried out using an automated system (AS-C3, Apollo Scitech), which was connected to an infrared gas detector (Li-Cor 7000, Li-Cor). Calculation of the carbonate system components (HCO_3_^−^, CO_3_^2−^, CO_2_ and TA) was carried out using known values of DIC, pH, nutrient concentration, salinity and temperature with a CO_2_ system analysis software (CO2SYS) [26] (Table 1). Carbonic acid dissociation constants (K_1_ and K_2_) were according to Roy et al. [27], and that for boric acid (K_B_) was taken from Dickson [28].

### Growth rate and cell size measurements

Cell numbers, mean cell volumes and size distributions were acquired with a Z2^TM^ Coulter Counter (Beckman, Buckinghamshire, UK). Determinations of growth rates were based on the cell number changes every 24 h and were calculated according to the equation: μ =  (ln*N*_1_–ln*N*_0_)/(*t*_1_–*t*_0_), where *N_1_* and *N_0_* are the cell concentrations before dilution (*t*_1_) and after the previous dilution (*t*_0_) respectively. Growth rates were calculated based on measurements of 11–12 replicates for triplicate cultures under each CO_2_ level.

### Carotenoid and Chlorophyll measurements

To determine the carotenoid and chlorophyll *a* and *c* contents of cells cultured in the different CO_2_ and NO_3_^−^ conditions, cells were collected by filtration on to Whatman GF/F filters (pore size, 0.22 μm) and extracted overnight with absolute methanol at 4°C. The extracts were then centrifuged for 10 min at 5000× g and the absorbance of the supernatant was scanned with a spectrophotometer (DU800, Beckman, Fullerton, California, USA). Calculation of chlorophyll *a* from the absorbance spectra followed the equation of Porra [29], chlorophyll *c* was after Ritchie [30] and carotenoid was calculated according to Strickland and Parsons [31].

### Quantum yield measurements

The quantum yield of cells grown in the different CO_2_ and NO_3_^−^ conditions was measured with a XE-PAM (Walz, Germany) at both mid-light phase (F_v_'/F_m_') and at the end of the dark phase (F_v_/F_m_). The saturation light was set at 5000 μmol photons m^−2^ s^−1^ for 0.8 s.

### Determination of CCM activity from rETR vs DIC curves

To estimate the affinity of cells for DIC (used as a proxy for CCM activity), cells cultured in different CO_2_ and NO_3_^−^ conditions were collected, washed with, and re-suspended into, DIC-free seawater with a pH of 8.20. Cell densities after re-suspension were between 3 and 4×10^4^ cells ml^−1^. The DIC-free seawater was prepared by adding 1 mol L^−1^ HCl to drop the pH below 3 and then bubbling with pure N_2_ for 1 h. Tris-buffer was added to 20 mmol L^−1^ to adjust the pH back to 8.20. Cells suspended in the DIC- free seawater were incubated at 150 µmol m^−2^ s^−1^ for 15 min to exhaust any intracellular DIC, and NaHCO_3_ solution was then added into each vial of algal suspension to obtain different DIC concentrations. After further incubation under a photon flux of 70 µmol m^−2^ s^−1^ for 10 min (less than 0.1% DIC was consumed), a rapid light curve was determined with the XE-PAM, and the resulting data fitted with the equation of Eilers and Peeters [32]: y = x/(ax^2^+bx+c), where a, b, c are estimated parameters, x photon flux density, and y the rETR value. Light saturated rates of electron transport (rETR_max_) at the different DIC concentrations can be calculated from the fitted rapid light curve: rETR_max_ = 1/[b+2(ac)^1/2^], and the light harvesting efficiency (α) was calculated with the equation: α = 1/c. To quantify the relationship between rETR_max_ and DIC concentrations, we fitted the two parameters using the Michaelis-Menten equation to determine light- and DIC-saturated rates of photosynthesis and the half-saturation constant K_1/2 DIC_ and K_1/2 CO2_ for DIC-dependent electron transport.

### Dark respiration measurements

Cells were gently filtered on to polycarbonate membrane filters (0.22 μm, Q/YY8-1-88, Xinya, China) with a vacuum pump at a pressure of less than 0.02 Pa. to ensure cells were intact, based on a microscopic checkup, and were then re-suspended into 20 mmol L^−1^ Tris-buffered media of the respective composition (LC-HN, LC-LN, HC-HN and HC-LN). Each treatment had a known cell concentration of around 1×10^6^ cells ml^−1^. Dark respiration rates were determined with a Clark type oxygen electrode (5300A, YSI) from changes in oxygen concentration over time at 20°C. A two-point calibration (seawater bubbled with air until equilibrium saturation and O_2_ deprivation with excess sodium sulfite as zero oxygen) was carried out before respiration measurements. The possible contribution of bacterial respiration was tested on the culture filtrate that passed through a filter pore size of 1μm (mixed cellulose lipid membranes), which would not exclude the few bacteria present, and there was no detectable bacterial O_2_ consumption.

### Measurement of carbon and nitrogen contents

To determine particulate organic carbon (POC) and nitrogen (PON) in *Phaeodactylum tricornutum* grown under different CO_2_ and NO_3_^−^ concentrations, cells were collected in the mid-light period by filtration onto pre-combusted (450°C, 6 h) GF/F filters (Whatman). Filters were acidified with 0.1N HCl fumes for 12 h and then dried overnight in an oven at 60°C. Carbon and nitrogen contents were determined with a PerkinElmer Series II CHNS/O Analyzer 2400.

### Statistical analysis

One-way analysis of variance (ANOVA) and Tukey's test were used to establish differences among treatments at a confidence level of 95%. Interactive effects between CO_2_ and NO_3_^−^ were analyzed using a Tukey post hoc test.

## Results

### Growth rate

**Figure 1:**
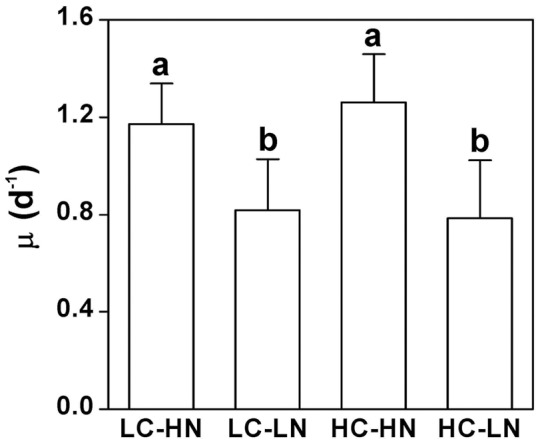
Specific growth rates of *P. tricornutum*. The growth rates were measured after cells acclimated for 10 generations under nitrogen limited (LN) and replete (HN) levels in 390 (LC) and 1000 μatm (HC) CO_2_ conditions. The different letters indicate significant differences among the treatments at the *P*<0.05 level. Vertical bars are means ±SD, n = 11–12.

Growth rates were inhibited under nitrogen limited conditions under both LC (30%, *P*<0.001) and HC (38%, *P*<0.001) conditions (Figure 1). No direct effects on growth rate were found between the CO_2_ treatments (nitrogen replete, *P* = 0.24; nitrogen limited, *P* = 0.72). No interactive effect was found between CO_2_ and NO_3_^−^ levels (*P* = 0.3).

### Chlorophyll and carotenoid contents

**Figure 2:**
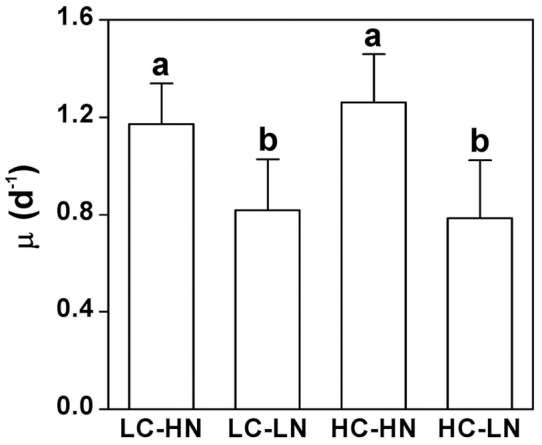
Pigments of *P. tricornutum*. (a) Carotenoid, (b) chl *a*, (c) chl *c* contents and (d) ratio of chl *a* to carotenoid of *Phaeodactylum tricornutum* grown at nitrogen limited (LN) and replete (HN) levels in 390 (LC) and 1000 μatm (HC) CO_2_ conditions, measured after the cells had acclimated for 10 generations. The different letters indicate significant differences among the treatments at the *P*<0.05 level. Vertical bars are means ±SD, n = 9–13.

The effects of nitrogen and CO_2_ treatments on cellular chlorophyll and carotenoid concentrations showed the same trend as cell size and growth rate. Nitrogen limitation decreased the carotenoid, chlorophyll *a* and *c* contents by 50% and 62% (Figure 2A), 48% and 60% (Figure 2B), 45% and 63% (Figure 2C) in the LC and HC groups, respectively (*P*<0.05). No direct effects on pigmentation were found between the CO_2_ treatments (*P*>0.05). The ratio of chlorophyll *a* to carotenoids showed no significant difference among the treatments (*P*>0.05) (Figure 2D). No interactive effects on chlorophyll *a* (*P* = 0.086), *c* (*P* = 0.133), carotenoid (*P* = 0.475) and the ratio of chlorophyll *a* to carotenoids (*P* = 0.657) were found between CO_2_ and NO_3_^−^ levels.

### Quantum yield

**Figure 3:**
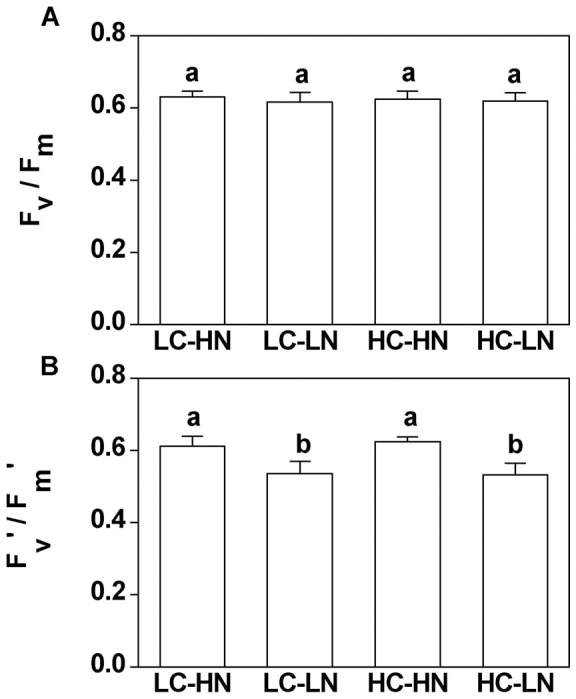
Photochemical quantum yield of *P. tricornutum*. (a) The maximal (F_v_/F_m_) and (b) effective (F_v_'/F_m_') quantum yield of *P. tricornutum* cells grown in LC-HN, LC-LN, HC-HN and HC-LN conditions, measured after the cells had acclimated for 10 generations. The different letters indicate significant differences between treatments at the *P*<0.05 level. Vertical bars are means ±SD, n = 3–5.

Maximum quantum yields (F_v_/F_m_), measured at the end of the dark period, showed no significant differences among treatments (*P*>0.05) (Figure 3A). However, in the mid-light period, cells cultured under nitrogen limited conditions showed decreases in effective quantum yield (F_v_'/F_m_') by 12% and 15% in the LC (*P* = 0.01) and HC (*P* = 0.002) treatments respectively, compared to those under nitrogen replete conditions (Figure 3B). No significant (nitrogen replete, *P* = 0.43; nitrogen limited, *P* = 0.91) change in the yield was found between the low and high CO_2_ levels (Figure 3A, B). No interactive effect on yield was found between CO_2_ and NO_3_^−^ levels (F_v_/F_m_, *P* = 0.24; F_v_'/F_m_', *P* = 0.58).

### Cell size

**Figure 4:**
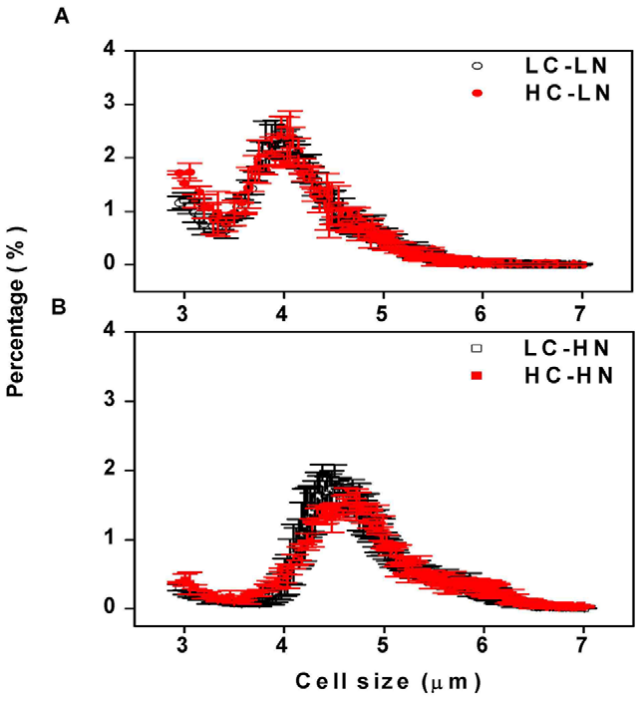
Cell sizes of *P. tricornutum* . The cells grown at (a) N-limited (LN) and (b) N-replete (HN) conditions with 390 and 1000 μatm CO_2_, measured after the cells had acclimated for 10 generations. Vertical bars are means ±SD, n = 2–4.

**Figure 5:**
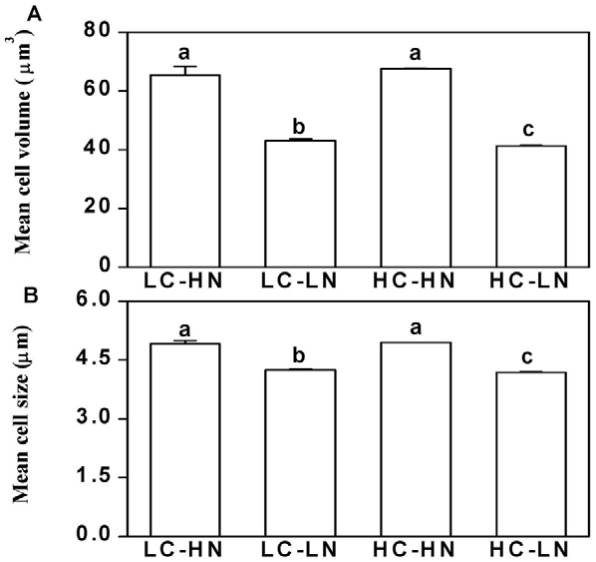
Mean cell volume and size of ***P. tricornutum***. (a) Mean cell volume and (b) cell size of *Phaeodactylum tricornutum* cultured under N-limited and N-replete conditions with 390 and 1000 μatm CO_2_, measured after the cells had acclimated for 10 generations. Vertical bars are means ±SD, n = 2–4.

Increased dissolved CO_2_ concentration did not affect the mean cell size (as Effective Spherical Diameter) and cell volume of *Phaeodactylum tricornutum* under nitrogen replete conditions. However, nitrogen limitation did cause significant (*P*<0.001) decreases in cell size and cell volume (Figure 4, 5). Mean cell volumes were 65.3 and 67.6 µm^3^ in LC and HC treatments, and nitrogen limitation significantly decreased these values to 43.0 and 41.3 μm^3^ (by 34% and 39%), respectively (*P*<0.001) (Figure 5A). Mean cell sizes (as Effective Spherical Diameter) in LC and HC were 4.91±0.08 and 4.95±0.00 µm respectively, under nitrogen replete conditions (Figure 5B). Under nitrogen limitation, cell size decreased by 13% and 15% (*P*<0.001), to 4.25±0.02 µm and 4.19±0.01 µm in LC and HC cells, respectively (Figure 5B). No significant differences in both cell size (*P* = 0.56) and volume (*P* = 0.39) were found between low and high CO_2_ levels under the nitrogen replete conditions. However, high CO_2_ cells showed a small but significant decrease of both cell size (*P* = 0.02) and volume (*P* = 0.04) when nitrogen was limited. No interactive effects were found between CO_2_ and NO_3_^−^ levels in both cell size (*P* = 0.16) and cell volume (*P* = 0.14).

### P vs DIC characteristics

**Figure 6:**
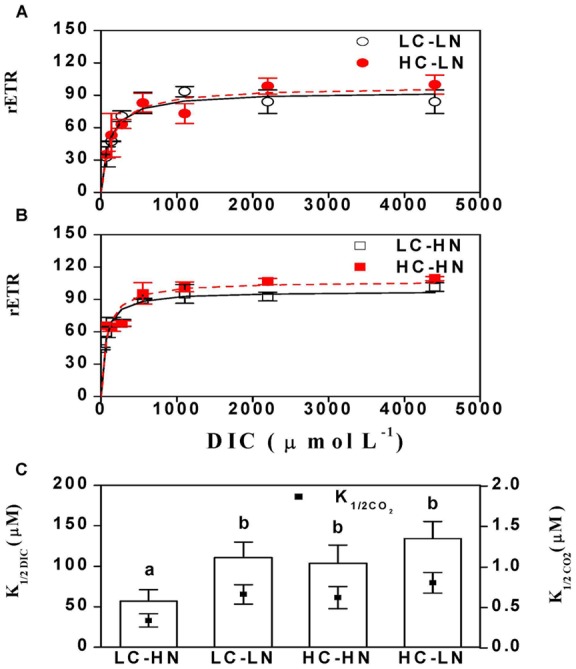
Photosynthetic electron transfer rate, K_1/2 DIC_ or K_1/2 CO2_ of *P. tricornutum*. Photosynthetic electron transfer rate of the cells cultured in (a) HC-HN, HC-LN and (b) LC-HN and LC-LN when measured at different DIC concentrations. (c) K_1/2 DIC_ or K_1/2 CO2_ values were calculated from (a) and (b). The different letters indicate significant differences between treatments at the *P*<0.05 level. Vertical bars are means ±SD, n = 3 (except LC-LN and HC-LN were 2 replicates).

**Figure 7:**
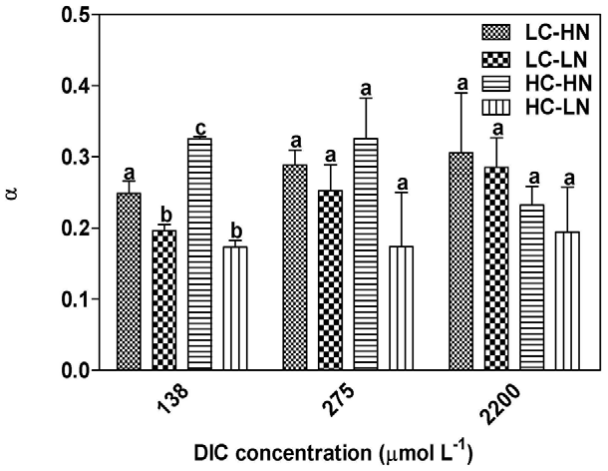
The apparent light use efficiency of *P. tricornutum*. The apparent light use efficiency of the cells cultured in LC-HN, LC-LN, HC-HN and HC-LN, when measured at DIC-limited or ambient levels. The different letters indicate significant differences among the treatments at the *P*<0.05 level. Vertical bars are means ±SD, n = 3 (except LC-LN and HC-LN were 2 replicates).

With increasing DIC concentration in the medium, high CO_2_, N-replete cultures had a significantly (p = 0.04) elevated rETR_max_ (light and DIC-saturated rate of electron transfer) compared to low CO_2_, N-replete cells (rETR_max_ values were HC-HN 110.08±5.78, LC-HN 97.47±4.55). The rETR_max_ under N-limited conditions was not affected by the CO_2_ level (LC-LN (n = 2) 93.24±2.12, HC-LN (n = 2) 98.31±7.99; *P* = 0.48) (Figure 6A, B). The calculated K_1/2 DIC_ values indicate that CCM activity was down-regulated under HC conditions (*P* = 0.04), with K_1/2 DIC_ values in the high N-grown cells increasing from 57.0 (LC-HN) to 103.4 µmol L^−1^ (HC-HN). N-limitation also caused a rise in K_1/2 DIC_ to 110.4 µmol L^−1^ even under low CO_2_, and this increased to 134.2 µmol L^−1^ in HC-LN cells (Figure 6C). The K_1/2 CO2_ shows the same trend as K_1/2 DIC_ and values were 0.34, 0.66, 0.62 and 0.81 µmol L^−1^ in the LC-HN, LC-LN, HC-HN and HC-LN treatments respectively. The light harvesting efficiency (α) of the cells, derived from RLC at different DIC levels, was not affected (*P* = 0.12) by CO_2_ levels when nitrogen was limited, but was significantly (*P* = 0.01) elevated by the CO_2_ enrichment in N-replete conditions at a DIC level of 138 μmol L^−1^. Such a trend was still observed at 275 μmol L^−1^, although with the differences being statistically insignificant (nitrogen replete, *P* = 0.35; nitrogen limited, *P* = 0.32) (Figure 7). At the ambient DIC level of 2200 μmol L^−1^, no significant difference (nitrogen replete, *P* = 0.22; nitrogen limited, *P* = 0.23) in light harvesting efficiency was found among the treatments (Figure 7).

### Dark respiration rates

Cells grown under nitrogen limitation, irrespective of CO_2_ level, showed approximately a doubling in respiration rate, compared to ambient CO_2_, nitrogen replete cells. With N-replete cells, high CO_2_ also resulted in an increase in cellular respiration rates (Figure 7A). When dark respiration was expressed on a per chlorophyll *a* basis, rates were enhanced by 298%, 110% and 305% in LC-LN, HC-HN, HC-LN treatments respectively, compared to the LC-HN conditions (Figure 7B). No interactive effects on dark respiration were found between CO_2_ and NO_3_^−^ levels (*P* = 0.16).

### Carbon and nitrogen contents

Nitrogen limitation significantly decreased the nitrogen content of cells cultured in both LC (by ca. 32%) and HC (by ca. 28%) conditions compared with the nitrogen-replete treatments (*P*<0.001) (Table 2). Nitrogen limitation led to an increase in the C:N ratio by 21% in the LC condition, and this enhancement increased to 45% in the HC condition (*P*<0.001). However, under the nitrogen replete treatment the CO_2_ concentration did not affect the C:N ratio (*P* = 0.24), even though the cells at the high CO_2_ level significantly increased their nitrogen content by 13% (*P* = 0.04). Turkey's post hoc test showed that there were significant interactive effects on C:N found between CO_2_ and NO_3_^−^ levels (*P*<0.001).

**Table 2:** Elemental stoichiometry of organic carbon and nitrogen contents and their ratios in *Phaeodactylum tricornutum*.

	C (pg cell^−1^)	N (pg cell^−1^)	C/N (mol/mol)
LC-HN	8.00±1.16^ a^	1.69±0.26^ a^	5.53±0.35^ a^
LC-LN	6.55±0.52^ b^	1.15±0.13^ b^	6.71±0.60^ b^
HC-HN	8.58±0.91^ ac^	1.91±0.13^ c^	5.26±0.56^ a^
HC-LN	9.29±0.82^ c^	1.37±0.24^ d^	8.02±0.69^ c^

The particulate organic carbon (POC), particulate organic nitrogen (PON) and molar ratio of POC to PON of *Phaeodactylum tricornutum* when grown in LC (390 μatm, LC) and HC (1000 μatm, HC) under NO_3_^−^ replete (HN) and limited (LN) conditions. Data are the means ±SD of 8 to 12 measurements. The superscripts of lowercase letters represent significant differences (*P*<0.05) between treatments.

## Discussion

**Figure 8:**
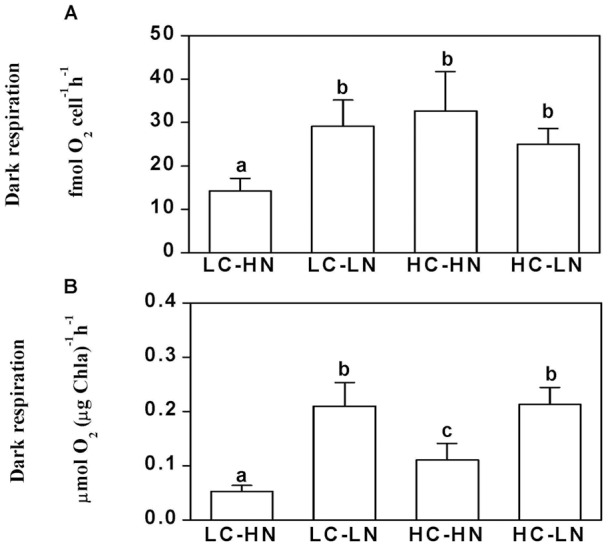
Dark respiration rates of *P. tricornutum*. Dark respiration rates (a) per cell or (b) per chl *a* cultured in LC-HN, LC-LN, HC-HN and HC-LN grown cells. The different letters above the bars indicate significant differences among the treatments at the *P*<0.05 level. Vertical bars are means ±SD, except the HC-LN treatments that had 2 replicates, all other data are from 4 replicates.

While the elevated CO_2_ concentration of 1000 μatm did not cause significant differences in growth, pigment contents, effective quantum yield and cell size, nitrogen limitation decreased all these parameters in the diatom *Phaeodactylum tricornutum* (Figures 1, 2, 3, 4). Both the elevation of CO_2_ and N-limitation led to a down-regulation of CO_2_ concentrating mechanism (CCM) activity, as reflected in the increased/decreased K_1/2 DIC_ or K_1/2 CO2_. N-limitation and increased pCO_2_/reduced pH led to the lowest light use efficiency under Ci-limited conditions (Figure 7), with this trend effect being minimized under elevated levels of DIC. Ocean acidification increased dark respiration under N-limited conditions (Figure 8). The high CO_2_ and low nitrate combination altered the cells' elemental stoichiometry, with the C:N ratio increased by 45% compared to the low CO_2_ and nitrate replete grown cells (Table 2).

### Basic cell parameters

Confirming previous studies on growth of diatoms, including *P. tricornutum*, under elevated CO_2_ [33], [34], growth at the elevated CO_2_ levels that are expected by the end of the century did not cause a significant increase in growth rate. This was the case regardless whether the organism was grown under N depletion or N repleted conditions. Earlier studies in our laboratory on the same species showed an enhanced growth rate (ca. 5%) under elevated CO_2_ and a PAR of 120 μmol m^−2^ s^−1^ [11]. In the current study we did not find improved growth under a PAR level of 70 μmol m^−2^ s^−1^. Recently, changes in light levels have recently been shown to mediate diatoms' responses to ocean acidification [14]. Even although both light levels are sub-saturating for photosynthesis, less photosynthetic carbon fixation under 70 μmol m^−2^ s^−1^ should have resulted in less or no growth stimulation due to the enhanced respiratory carbon loss (Figure 8).

Although growth rates were clearly limited by a decrease in N-supply and, as has commonly been reported [35], chlorophyll levels were significantly decreased in N-limited cells, there was no change in the chl:carotenoid ratio, sometimes used as an indicator for N-limitation [35], under our experimental conditions (Figure 2). A lack of effect of CO_2_ levels on cellular pigment content is consistent with previous data on diatoms [11], [36] and other microalgae [37]. Furthermore, dark-adapted maximal quantum yield showed no decrease in N-limited cells, although the effective quantum yield show a small, but significant drop; neither parameter was affected by growth at elevated CO_2_ (Figure 3). Previous studies on a green alga *Ulva rigida* showed that both F_v_/F_m_ and F_v_'/F_m_' were significantly down-regulated by high CO_2_, and that nitrogen limitation further decreased both parameters [38]. A decline in F_v_/F_m_ is a general response to nitrogen limitation [39]. There is a possibility that the urea cycle found in *Phaeodactylum* functions to support the photosynthetic machinery by recycling N under the N-limited conditions [40]. In the light period, in contrast, carbon fixation and nitrogen acquisition are both competing for energy [20], thereby leading to lower yield under the N-limited conditions.

Cell size was significantly smaller by ca. 15% in N-limited cells, a phenomenon that has been reported for dinoflagellates and for the coccolithophore, *Emiliania huxleyi* [41], [42], [43]. Considering the lower specific growth rate and increased C:N ratio under future high CO_2_ and low nutrient conditions (Table 2), the diatoms' carbon fixation efficiency per unit nitrogen will increase in the surface seawater.

### Photosynthesis vs DIC characteristics

Elevated CO_2_ caused a slight increase in photosynthetic capacity (measured as rETR_max_ under light and DIC-saturated conditions) under N-replete, but not under N-limited conditions (Figure 6). Small increases in the DIC-saturated photosynthetic capacity of diatoms grown under elevated CO_2_ have been reported previously [11], [36], [44]. Growth under N-limited conditions caused an increase in K_1/2 DIC_ in low-CO_2_ grown cells. This indicates a down-regulation of CCM activity by nitrogen limitation. This is in contrast to the reports of Young and Beardall for the green alga *Dunaliaella tertiolecta* showing cells grown under low CO_2_ exhibited increasing affinity (lower K_1/2__DIC_) for DIC under N-limited growth and work on *Chlorella emersonii* in which cells under 5% CO_2_ showed partial induction of CCM activity when N-limited [45], [46]. Such responses have been viewed as a way of improving N-use efficiency and maintaining Rubisco activity with less Rubisco protein when resources such as nitrogen are in short supply [45]. However, induction of CCM activity under N-limitation does not always occur, especially when cells are grown under low CO_2_ or are not severely N-limited (see Table 1) [39], [47]. In *P. tricornutum*, nitrogen storage strategy using the urea cycle could mediate the diatom's CCM to decrease the influence of elevated CO_2_ levels [40].

The changes in rETR_max_ under elevated CO_2_ and N-replete conditions may be partially negated by the rise in dark respiration and contribute to the lack of effect of high CO_2_ on growth rate. However, low CO_2_ grown cells that were N-limited also exhibited higher respiration rates, contributing to the lower growth rates found in N-limited cells. Growth at elevated CO_2_ exposes cells to a lower pH, which might impose additional energetic costs for acid-base regulation to sustain metabolic integrity [48]. Metabolic processes also influence the pH in the immediate proximity to the cell surface [49], and under low N levels (here supplied as nitrate), the near cell pH would become less alkaline (NO_3_^−^ uptake leads to OH^−^ extrusion), again leading to increased energy demand to maintain pH gradients across the cell membrane.

### Elemental composition

The elemental composition and macromolecular composition of phytoplankton is critically important for secondary producers such as copepods, fish and shrimp, and food nutritional quality influences energy flow through marine food chains [50]. Recently Rossoll et al. found that the fatty acid composition of the diatom *Thalassiosira pseudonana* cultured at elevated CO_2_ was altered and that this significantly affected the growth and egg production of a copepod, *Acartia tonsa* [51]. Riebesell et al. reported an increased C:N ratio in a mesocosm study dominated by diatoms [52], whereas Burkhardt et al. reported both increases and decreases in C:N ratio with increasing CO_2_, dependent on the species [23]. In our study, nitrogen limitation decreased both the carbon and nitrogen contents per cell but these changes were not parallel and led to an enhanced C:N ratio in both LC and HC conditions. Cells cultured under high CO_2_ showed increased cell quotas for both carbon and nitrogen, irrespective of nitrogen supply (Table 2). The highest C:N was found in the high CO_2_/low nitrogen conditions that are expected to dominate the open ocean in the near future, indicating that these synergistic effects of ocean acidification and nitrogen limitation could decrease the food quality of marine phytoplankton.

Although increased marine dissolved CO_2_ may bring some benefits in terms of improved carbon supply to some phytoplankton [8], these organisms also face an extra cost associated with changed marine chemistry, especially pH stress, which could also lead to more energetic constraints on growth. Thus, the net benefit of higher CO_2_ will be a balance between gains and losses determined by the various environmental factors associated with climate change.

In conclusion, ocean acidification together with ocean change can act in the marine environment synergistically or antagonistically to affect diatom performance, depending on the levels of sunlight [14]. Intensified stratification may push the marine phytoplankton into nitrogen-limited status, and will thereby influence the physiological or biochemical characteristics of the phytoplankton cells. Increases in respiratory metabolism may counteract any increase in the rate of C gain through photosynthesis (and hence affect net growth) [11], [14], [36]. Enhanced C:N ratios, induced by high CO_2_ and low nitrogen, can influence secondary producers as well as predators at higher levels. Effects of ocean acidification on marine primary producers can be species-specific due to their physiological diversities and vary between different oceanic regions correlated with differed physical, chemical or biological conditions.
