# Correction: Interactive Effects of Ocean Acidification and Nitrogen-Limitation on the Diatom *Phaeodactylum tricornutum*

**DOI:** 10.1371/annotation/98908e14-e9fd-458f-9cea-ba4bec139f20

**Published:** 2013-08-05

**Authors:** Wei Li, Kunshan Gao, John Beardall

Figure 2 is currently a duplicate of Figure 1. The legend is correct. Please see the correct version of Figure 2 at the link following the Figure 2 legend.

Pigments of P. tricornutum.

(a) Carotenoid, (b) chl a, (c) chl c contents and (d) ratio of chl a to carotenoid of Phaeodactylum tricornutum grown at nitrogen limited (LN) and replete (HN) levels in 390 (LC) and 1000 μatm (HC) CO2 conditions, measured after the cells had acclimated for 10 generations. The different letters indicate significant differences among the treatments at the P<0.05 level. Vertical bars are means ±SD, n = 9–13.

**Figure:**
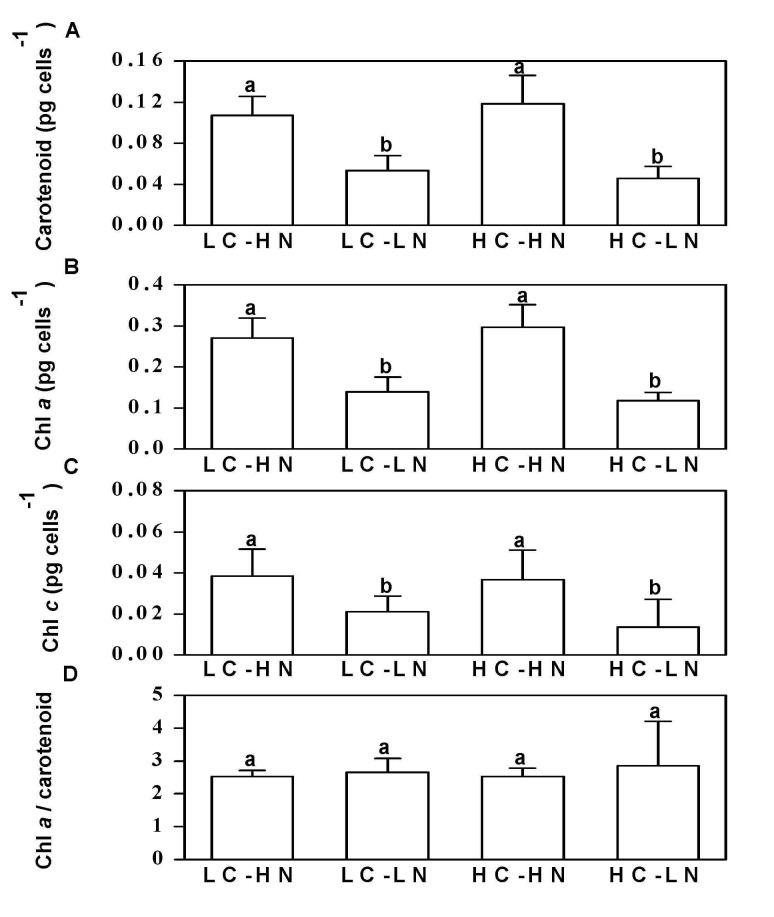


In the subheading Dark respiration rates, under the Results section, two in-text citations are incorrect: With N-replete cells, high CO2 also resulted in an increase in cellular respiration rates (Figure 7A). When dark respiration was expressed on a per chlorophyll a basis, rates were enhanced by 298%, 110% and 305% in LC-LN, HC-HN, HC-LN treatments respectively, compared to the LC-HN conditions (Figure 7B).

The citations are 8A and 8B, respectively. 

